# Yield of chest computed tomography angiogram in cystic fibrosis patients with suspected pulmonary embolism

**DOI:** 10.1111/crj.13473

**Published:** 2022-01-21

**Authors:** Kathleen Suzanne Mahan, Hamna Ahmad, Andrew George Keenan, Matthew Erren Prekker, Robert Ralph Kempainen

**Affiliations:** ^1^ Division of Pulmonary, Allergy, Critical Care and Sleep Medicine University of Minnesota, Twin Cities Minneapolis Minnesota USA; ^2^ Division of Pulmonary, Allergy, and Critical Care Medicine Hennepin Healthcare Minneapolis Minnesota USA

**Keywords:** cystic fibrosis, diagnostic imaging, disease exacerbation, pulmonary embolism

## Abstract

**Introduction:**

Individuals with cystic fibrosis (CF) may be at increased risk of pulmonary embolism (PE). Symptoms of PE overlap substantially with those of CF respiratory exacerbations. CF patients commonly undergo chest computed tomography (CT) angiograms (CTPA) to evaluate for PE, but little is known about the clinical presentation and diagnosis of PE in this population.

**Objectives:**

The objectives of this study are to determine the diagnostic yield of CTPA for PE in adult patients with CF and assess the utility of the Revised Geneva Score (RGS) in this population.

**Methods:**

Retrospective review of all CTPA results was performed on CF patients with suspected PE at a large CF center from 1 January 2011 through 31 March 2017. Patient demographics, medical history, and presenting signs and symptoms were abstracted by chart review.

**Results:**

A total of 103 unique CTPA studies were performed in 68 patients. Most were hospitalized at the time of CTPA, predominantly for respiratory manifestations of CF. CTPA identified four patients with PE. The small number of positive studies precluded analysis of predictors of PE. Fewer PE were diagnosed than predicted by the Revised Geneva Score, which was intermediate probability in 77/103 (75%) patients.

**Conclusion:**

The prevalence of PE in CF patients undergoing CTPA for suspected PE was 4%, which is lower than predicted by the Revised Geneva Score. This may be due to a large overlap in the signs and symptoms of PE and exacerbations of CF lung disease.

## 1 INTRODUCTION

Respiratory disease remains the primary source of morbidity and mortality in individuals with cystic fibrosis (CF), and each year, approximately 40% of adult patients experience a severe respiratory exacerbation requiring intravenous antibiotics.^1^ Pulmonary embolism (PE) is often a diagnostic consideration in CF patients undergoing evaluation for exacerbation as there is substantial overlap in signs and symptoms, including dyspnea, pleuritic chest pain, hemoptysis, tachycardia, and low‐grade fever. Furthermore, individuals with CF may be at greater risk of PE due to thrombophilia associated with lung inflammation, deficiency of anticoagulant factors from vitamin K deficiency or CF‐related liver disease, reduced mobility during illness, and use of central venous catheters (CVCs).^2^, ^3^, ^4^, ^5^ Numerous validated criteria can be used to calculate the pretest probability of PE in symptomatic patients, but these scoring systems are potentially less applicable to CF patients, especially in the setting of advanced lung disease and/or hospital admission.

The presence of overlapping signs and symptoms, risk factors for PE, and difficulty determining the pretest probability of PE may increase clinicians' reliance on chest computed tomography (CT) angiograms (CTPAs) to evaluate CF patients with possible venous thromboembolism. Although catheter‐based angiography was historically the gold standard, CTPA is now the first‐line diagnostic option for PE in the general population, with excellent sensitivity and specificity for proximal and segmental PE.^6^, ^7^, ^8^ Although PE is a potentially life‐threatening condition that merits thorough evaluation, the risks of kidney injury in CF patients receiving nephrotoxic antibiotics, as well as the cumulative risk of radiation exposure, particularly in young females, point toward the need to limit unnecessary use of CTPA.^9^ A large single‐center study identified a total of four PE among 458 children with CF hospitalized over a 13‐year period.^3^ Other studies looking at the incidence of thrombosis specifically among patients with totally implantable vascular access devices described infrequent observations of embolism.^10^, ^11^ A retrospective study from a large adult CF clinic found deep venous thrombosis (DVT) in 4.5% of 376 peripherally inserted central catheters (PICCs) during a 6‐year period, but the presence or absence of PE was not noted.^12^

These studies suggest PE is relatively uncommon. However, little is known about the incidence, clinical presentation, diagnostic evaluation, and treatment of PE in the CF population. To our knowledge, there are no previous reports of the yield of CTPA in consecutive CF patients with suspected PE. We evaluated the electronic medical records (EMRs) of all CF patients who underwent CTPA for possible PE between 2011 and 2017. The aims of this study were to determine the diagnostic yield of CTPA for PE in adult patients with CF; summarize the clinical presentation of PE; and assess the utility of the Revised Geneva Score (RGS), a validated tool for determining the pretest probability of PE, in the adult CF population.

## 2 MATERIALS AND METHODS

We performed a retrospective chart review of all CTPA studies performed on adult CF patients with suspected PE at the University of Minnesota CF Center from 1 January 2011 through 31 March 2017. The study procedures were approved by the University of Minnesota Institutional Review Board. The University of Minnesota Adult CF Center treats approximately 400 individual patients annually. The electronic medical record was queried for all patients 18 years of age or older with a diagnosis of CF that underwent a chest CT scan. Non‐CTPA scans, CTPA scans obtained in lung transplant recipients, and CTPA studies performed for purposes other than PE evaluation (e.g., prior to bronchial arterial embolization) were excluded. CTPA results, as well as patient demographics, medical history, presenting signs and symptoms, and presence or absence of risk factors for PE were extracted via detailed chart review. PE risk factors included for chart review included age, use of oral contraception or other hormone replacement, cigarette smoking, recent major trauma or surgery, active malignancy, prior history of venous thromboembolism, body mass index (BMI), diabetes, current or recent pregnancy, and presence of CVC.^13^

We utilized the RGS to retrospectively calculate the pretest probability of PE using characteristics present at the time of CTPA acquisition for each patient.^14^ The nine characteristics included in scoring are as follows: age >65 (1 point), prior DVT or PE (3 points), surgery under general anesthesia or lower limb fracture within 1 month (2 points), active malignant condition (2 points), unilateral lower limb pain (3 points), hemoptysis (2 points), heart rate 75–94 beats per minute (3 points), heart rate ≥95 beats per minute (5 points), pain on lower‐limb deep venous palpation, and unilateral edema (4 points). Low, intermediate, or high probability scores for PE are 0–3, 4–10, and >10, respectively. The original validation study of the RGS found an incidence of 8% in the low probability group, 29% in the intermediate probability group, and 74% in the high probability group.^14^

RGSs were calculated using STATA software (StataCorp, College Station, TX). GraphPad Prism (GraphPad, San Diego, CA) statistical software was used. Redcap (Vanderbilt University, Nashville, TN) was used for data management and descriptive statistics.

## 3 RESULTS

During the approximately 5‐year study period, a total of 112 CTPA scans were performed on adult CF patients. Nine encounters were excluded due to prior lung transplantation or because the CTPA was obtained in preparation for bronchial arterial embolization rather than suspicion of PE. A total of 103 CTPA studies obtained from a total of 68 patients were included in the analysis. There were four CTPA studies (4%) positive for PE. Characteristics of patients with and without positive CTPA studies are summarized in Table 1. The overall patient population was young (median age 31 years), and the vast majority (84%) were hospitalized at the time of the CTPA, most commonly for an exacerbation of lung disease. Forty‐one percent had a CVC at the time of CTPA acquisition. The small number of positive studies precluded analysis of predictors of PE.

**TABLE 1:** Characteristics of patients undergoing chest CT angiogram

	Pulmonary embolism (*n* = 4)	No pulmonary embolism (*n* = 99)
Age, years, median (IQR)	24 (22–25)	31 (24–41)
Male sex, *N* (%)	0	39 (39)
DeltaF508 status, *N* (%)		
Homozygous	0	49 (50)
Heterozygous	3 (75)	31 (31)
Other	1 (25)	19 (19)
BMI, mean (*SD*)	18.8 (2.7)	21.8 (4.4)
Diabetes, *N* (%)	3 (75)	54 (55)
Best FEV_1_ (L), median (IQR)^a^	1.53 (1.29–2.15)	1.81 (1.33–2.59)
Inpatient status, *N* (%)	4 (100)	82 (83)
Central venous catheter, *N* (%)	3 (75%)	38 (40)
CVC type, *N* (%)		
PICC	1 (25)	19 (50)
Port‐a‐cath	2 (50)	18 (47)
Central line (nontunneled)	0	1 (3)
History of VTE	3 (75)	18 (18)
Hormone therapy, *N* (%)	1 (25)	18 (18)
Current smoker	0	2 (2)
Admission diagnosis		
CF exacerbation	3 (75)	57 (58)
Pneumonia	0	11 (11)
Hemoptysis	0	8 (8)
Other (SBO, pleuritis, and RUQ pain)	1 (25)	23 (23)
Revised Geneva Score		
Mean (*SD*)	8.5 (3.3)	5.4 (2.3)
Median (IQR)	8 (6.5–10.5)	5 (5–7)
Symptoms at time of CTPA, *N* (%)		
Hemoptysis	2 (50)	24 (24)
Dyspnea	0	33 (33)
Chest pain	2 (50)	41 (41)
Other	2 (50)	26 (26)
D‐dimer, *N* (%)		
Not measured	4 (100)	83 (84)
Normal		6 (6)
Elevated		10 (10)

Abbreviations: BMI, body mass index; CF, cystic fibrosis; CTPA, chest CT angiogram; CVC, central venous catheter; FEV_1_, forced expiratory volume in 1 s; IQR, interquartile range; *N*, number; PICC, peripherally inserted central catheter; RUQ, right upper quadrant; *SD*, standard deviation; SBO, small bowel obstruction; VTE, venous thromboembolism.

^a^ Highest recorded FEV_1_ in the 6 months prior to chest CT angiogram.

RGSs for patients with and without a diagnosis of PE are presented in Table 2. Seventy‐five percent (*n* = 77) of all patients had a score indicating intermediate probability; two components of the RGS, primarily heart rate >94 and hemoptysis, both common findings in a CF exacerbation, contributed significantly to a score between 4 and 10 (Table 3). Nearly half of patients had a heart rate >94 and 33% had hemoptysis at the time of the CTPA study resulting in contributions of 5 and 2 points, respectively. The probability of PE was high at the time of the CTPA in only 2% of studies based on RGS scoring. Three of the four patients with a PE had intermediate scores (5, 8, and 8), and one had a high score (13). Of note, two of the three PE‐positive patients with intermediate probability RGS scores had a permanent CVC at the time of presentation, and the one patient with the high probability score did not have a CVC present. D‐dimer assay was obtained in 15.5% (*n* = 16) of patients and was elevated in 9.7% (*n* = 10) of those tested. The four individuals with PE did not undergo D‐dimer testing.

**TABLE 2:** Revised Geneva scores of patients undergoing chest CT angiogram

	PE (*n* = 4)	No PE (*n* = 99)	All (*n* = 103)
Geneva category			
Low risk (0–3)	0	21 (21%)	21 (20%)
Intermediate risk (4–10)	3 (75%)	77 (78%)	80 (78%)
High risk (≥11)	1 (25%)	1 (1%)	2 (2%)

Abbreviation: PE, pulmonary embolism.

**TABLE 3:** Revised Geneva scores of all patients

	Low (0–3)	Intermediate (4–10)	High (≥11)
Geneva category			
Heart rate			
<75	7	1	0
75–94	17	26	1
≥95	0	50	1
Hemoptysis	0	32	2
Age > 65	0	1	0
VTE history	1	19	1
Surgery or fracture in last month	1	1	0
Active malignancy	0	2	0
Unilateral leg pain	0	0	2
Pain with palpation, edema	0	2	2
Without PE (99)	24	74	1
With PE (4)	0	3	1
Total (103)	24	77	2

Abbreviations: PE, pulmonary embolism; VTE, venous thromboembolism.

Additional details on the demographics, presentation, diagnosis, and clinical course of the four patients with scans positive for PE are displayed in Tables 4 and 5. All four patients diagnosed with PE were female, three had severely or very severely reduced forced expiratory volume in 1 s (FEV_1_) at time of admission, three had a CVC, and three had a prior history of DVT, one of whom also had a prior PE. The international normalized ratio (INR) in each of these three patients was subtherapeutic (<2) at the time of the CTPA study. Three were hospitalized at the time of diagnosis. One patient did not survive hospitalization and suffered a cardiopulmonary arrest; the subsequent CTPA revealed only a small PE with unclear clinical significance. Of the three surviving patients, one had recurrent venous thromboembolism (VTE) which occurred 2 years after the original PE was identified.

**TABLE 4:** Characteristics of the four patients diagnosed with pulmonary embolism

	Patient 1	Patient 2	Patient 3	Patient 4
Age, sex	26, F	22, F	25, F	22, F
BMI	20	16	17	22
FEV_1_ (%)	63	43	32	41
Admit diagnosis	CFE	CFE	Abdominal pain	CFE and PE
Presenting symptoms	Hemoptysis and pleuritic chest pain	Dyspnea and cough	Cardiac arrest	Hemoptysis and pleuritic chest pain
CVC on admission	No	Yes (port)	Yes (PICC)	Yes (port)
DVT present	No	No	No	Yes
VTE history	Yes (DVT)	Yes (DVT)	Yes (DVT and PE)	No
Revised Geneva Score	13	8	8	5

Abbreviations: BMI, body mass index; CFE, cystic fibrosis exacerbation; CVC, central venous catheter; DVT, deep vein thrombosis; FEV_1_ (%), forced expiratory volume % predicted; PE, pulmonary embolism; PICC, peripherally inserted central catheter; VTE, venous thromboembolism.

**TABLE 5:** Treatment and clinical course of patients with pulmonary embolism

Patient	PE location	Treatment	Initial INR goal	Duration of treatment	Complication/recurrence
1	Small, nonocclusive in L main PA	Heparin bridge to coumadin	2–3	3 months	None
2	Distal right main extending into RML and RLL	Coumadin	2–2.5	Lifelong	Stenosis of the RUL and RML pulmonary arteries
3	Small, nonocclusive segmental and subsegmental RLL	Heparin			Death during admit from other causes
4	Small, nonocclusive segmental chronic clot LLL and RLL	Heparin bridge to coumadin	1.5–2	Lifelong	Upper extremity DVT then subsegmental PE post lung tx

Abbreviations: DVT, deep vein thrombosis; L, left; LLL, left lower lobe; PA, pulmonary artery; PE, pulmonary embolism; RLL, right lower lobe; RML, right middle lobe; RUL, right upper lobe; tx, transplant.

## 4 DISCUSSION

To our knowledge, this is the first published study to summarize the yield of CTPA in consecutive CF patients with suspected PE. Although multiple factors potentially place CF patients at especially high risk of PE,^2^, ^3^, ^4^, ^5^ the current analysis identified only four cases of pulmonary emboli in a large adult CF center over a more than 5‐year period. CTPA is the diagnostic modality of choice in suspected PE, and it is unlikely that the study failed to identify additional patients with PE. Also, noteworthy was the much lower prevalence of PE than was predicted using a validated scoring system for determining pretest probability. The overall 4% prevalence of PE among patients undergoing CTPA in the current study is lower than the 15–20% prevalence reported in major trials of CTPA.^6^, ^7^, ^8^ The relatively low yield of CTPA in this population is likely due to the substantial overlap in the presentation of CF respiratory exacerbation and that of PE. Approximately 29% of patients with an intermediate pretest probability of PE according to the RGS would be expected to actually have a PE.^14^ In the current study, nearly 80% of CTPA had an intermediate pretest probability of PE at the time of the study, but only 3.9% (3/77) of this group had a positive CTPA.

Elevation in the RGS in the CF population was driven primarily by tachycardia and the presence of hemoptysis, with age >65, malignancy, trauma, recent surgery, and signs and symptoms of DVT being uncommon. Half of patients received 5 points for heart rate >94 per minute, and approximately 33% of all patients received 2 points for hemoptysis. Acute worsening of dyspnea, pleuritic chest pain, and low‐grade fever are not part of the RGS scoring system but are common manifestations of CF lung disease that could also heighten the clinical suspicion for PE. Additionally, commonly utilized PE risk scoring systems do not include the presence of a CVC as a risk factor,^14^, ^15^, ^16^, ^17^ whereas multiple studies demonstrate their use increases the risk for DVT among individuals with CF.^2^, ^3^

The relatively low yield of CTPA in the study and limited ability of the RGS to stratify the risk of PE highlight the need for an improved means of determining the pretest probability of PE in CF patients. The study results suggest the presence of a CVC, severe lung disease, diabetes, prior history of venous thromboembolism, and female gender are potential risk factors especially relevant to CF patients, but a much greater number of events across multiple centers would be needed to derive and validate a pretest probability score specific to CF. As it stands, clinicians must weigh risks and benefits of CTPA relevant to the CF population when PE is a possibility. Cumulative radiation exposure is of greater concern in individuals with CF given their relatively young age and the chronic nature of the disease.^9^ Contrast‐induced nephropathy is another consideration, as individuals with CF are at increased risk of chronic renal disease due to drug toxicities, CF‐related diabetes mellitus, and nephrolithiasis.^18^ However, there is of course substantial morbidity and mortality associated with unrecognized PE.

The retrospective use of the RGS is a potentially important study limitation. The RGS is better suited for retrospective scoring because, unlike the Wells and modified Wells scores, it does not rely on clinicians' impression of the most likely diagnosis.^14^, ^19^, ^20^ However, it is possible that chart review did not as accurately capture all aspects of RGS scoring as prospective collection would offer. Another concern is that the RGS, like other prediction tools for DVT and PE, was derived and validated in outpatient settings, typically in emergency departments. However, current guidelines recommend the calculation of pretest probability even in hospitalized patients, and the limited studies of pretest probability scoring systems applied to hospitalized patients suggest the prevalence of DVT and PE may actually be greater among inpatients than predicted by pretest probability calculators.^18^, ^21^, ^22^, ^23^ Recent trials indicate age‐adjusted D‐dimer values can be used to identify patients with intermediate probability of PE that do not need CTPA studies, but this is less applicable to the current study given the overall young age of our patients.^24^, ^25^ The uncertain role of D‐dimer testing in patients with intermediate likelihood of PE during the 2011–2017 study period, as well as lower predictive value of D‐dimer testing in hospitalized patients, likely accounts for the small proportion of assays obtained in the study population.^26^ There is currently limited data on D‐dimer values in people with CF. Given the elevated inflammatory state of individuals with CF, particularly due to chronic bacterial infection, baseline D‐dimer may be abnormally high. Only 16 patients were tested for D‐dimer in this study; interestingly, four out of five tests in the low risk group were elevated, 6 out of 10 in the intermediate risk group were elevated, and the one patient tested in the high risk group was within normal limits. This area remains ripe for future research.

CTPA is the first‐line diagnostic choice for patients with suspected PE that cannot be excluded with the use of D‐dimer assays and pretest probability scores.^13^, ^21^ Our CF center's practice is to obtain CTPA for patients with an intermediate or high suspicion for PE unless there is an absolute contraindication to CTPA. However, it is possible the study did not identify a small subset of patients diagnosed with PE by ventilation‐perfusion scan or lower extremity ultrasound rather than CTPA during the study period. Poor quality CTPA studies or errors in radiologic interpretation could potentially contribute to the observed lower yield. However, none of the four patients diagnosed with PE had a negative CTPA at our center during the 3 months prior to the positive study. Furthermore, none of the patients with a negative CTPA were subsequently diagnosed with PE during the study period. This suggests that the observed low yield of CTPA is not due to false‐negative studies.

This was a single‐center study, and the threshold for obtaining a CTPA may vary across institutions. Of note, 80% of patients were intermediate or high risk for PE based on the RGS, which suggests obtaining CTPA was a reasonable decision for the vast majority of patients. As previously noted, a greater number of patients are needed to identify predictors of PE which would accurately stratify risk in the CF population.

In conclusion, compared with studies of suspected PE in other populations, PE was uncommon in individuals with CF and much lower than predicted. This is likely due to a large overlap between signs and symptoms of PE and manifestations of exacerbations of CF lung disease, primarily based upon the presence of tachycardia and hemoptysis. Based on this study, the ability of the RGS, and likely other pretest probability scores, to stratify the likelihood of PE may be limited. Use of a large, multicenter database might allow for the derivation and validation of a PE risk stratification tool specific to CF that would better identify the patients most suitable for CTPA testing.

## CONFLICT OF INTEREST

The authors have no potential conflicts of interest relevant to this study.

## AUTHOR CONTRIBUTIONS

K. M.: Literature search, data collection, study design, data analysis, manuscript preparation, and review of manuscript.

H. A.: Literature search, data collection, and review of manuscript.

A. K.: Literature search, data collection, and review of manuscript.

M. P.: Study design, data analysis, and review of manuscript.

R. K.: Literature search, data collection, study design, data analysis, manuscript preparation, and review of manuscript.

## ETHICS STATEMENT

The study protocol was approved by the Institutional Review Board of the University of Minnesota (IRB #1611M98921). The procedures followed were in accordance with the 1975 Declaration of Helsinki. This was a retrospective review of medical records, and identifying patient information was destroyed in the locked database. The authors retained a deidentified dataset.

## Data Availability

The data that support the findings of this study are available from the corresponding author upon reasonable request.

## References

[1] Cystic Fibrosis Foundation Patient Registry Annual Data Report. Bethesda, Maryland. 2018. <https://www.cff.org/Research/Researcher-Resources/Patient-Registry/2018-Patient-Registry-Annual-Data-Report.pdf>

[2] Takemoto CM . Venous thromboembolism in cystic fibrosis. Pediatr Pulmonol. 2012;47(2):105‐112. 22006666 10.1002/ppul.21566

[3] Knight‐Perry J , Branchford B , Thornhill D , Martiniano S , Sagel S , Wang M . Venous thromboembolism in children with cystic fibrosis: retrospective incidence and intrapopulation risk factors. Thromb Res. 2017;158:161‐166. 28934665 10.1016/j.thromres.2017.08.022

[4] Raffini L , Raybagkar D , Blumenstein M , Rubenstein R , Manno C . Cystic fibrosis as a risk factor for recurrent venous thrombosis at a pediatric tertiary care hospital. J Pediatr. 2006;148(5):659‐664. 16737881 10.1016/j.jpeds.2005.11.032

[5] Barker M , Thoenes D , Döhmen H , Friedrichs F , Pfannenstiel C , Heimann G . Prevalence of thrombophilia and catheter‐related thrombosis in cystic fibrosis. Pediatr Pulmonol. 2005;39(2):156‐161. 15633202 10.1002/ppul.20158

[6] van Belle A , Buller H , Huisman M , et al. Effectiveness of managing suspected pulmonary embolism using an algorithm combining clinical probability, D‐dimer testing, and computed tomography. Jama. 2006;295(2):172‐179. 16403929 10.1001/jama.295.2.172

[7] Anderson D , Kahn S , Rodger M , et al. Computed tomographic pulmonary angiography vs. ventilation‐perfusion lung scanning in patients with suspected pulmonary embolism: a randomized controlled trial. Jama. 2007;298(23):2743‐2753. 18165667 10.1001/jama.298.23.2743

[8] Righini M , Van Es J , Den Exter M , et al. Age‐adjusted D‐dimer cutoff levels to rule out pulmonary embolism: the ADJUST‐PE study. Jama. 2014;311(11):1117‐1124. 24643601 10.1001/jama.2014.2135

[9] Ferris H , Twomey M , Moloney F , et al. Computed tomography dose optimization in cystic fibrosis: a review. World J Radiol. 2016;8(4):331‐341. 27158420 10.4329/wjr.v8.i4.331 PMC4840191

[10] Munck A , Kheniche A , Alberti C , et al. Central venous thrombosis and thrombophilia in cystic fibrosis: a prospective study. J Cyst Fibros. 2015;14(1):97‐103. 25107684 10.1016/j.jcf.2014.05.015

[11] Aitken M , Tonelli M . Complications of indwelling catheters in cystic fibrosis: a 10‐year review. Chest. 2000;118(6):1598‐1602. 11115445 10.1378/chest.118.6.1598

[12] Nash E , Helm E , Stephenson A , Tullis E . Incidence of deep vein thrombosis associated with peripherally inserted central catheters in adults with cystic fibrosis. J Vasc Interv Radiol. 2009;20(3):347‐351. 19157904 10.1016/j.jvir.2008.11.018

[13] Konstantinides SV , Meyer G , Becattini C , et al. ESC guidelines for the diagnosis and management of acute pulmonary embolism developed in collaboration with the European Respiratory Society (ERS): the task force for the diagnosis and management of acute pulmonary embolism of the European Society of Cardiology (ESC). Eur Heart J. 2019;41(4):543‐603. 10.1093/eurheartj/ehz405 31504429

[14] LeGal G , Righini M , Roy P , et al. Prediction of pulmonary embolism in the emergency department: the Revised Geneva Score. Ann Intern Med. 2006;144(3):165‐171. 16461960 10.7326/0003-4819-144-3-200602070-00004

[15] Wells P , Anderson D , Rodger M , et al. Excluding pulmonary embolism at the bedside without diagnostic imaging: management of patients with suspected pulmonary embolism presenting to the emergency department by using a simple clinical model and D‐dimer. Ann Intern Med. 2001;135(2):98‐107. 11453709 10.7326/0003-4819-135-2-200107170-00010

[16] Kline J , Mitchell A , Kabrhel C , Richman P , Courtney D . Clinical criteria to prevent unnecessary diagnostic testing in emergency department patients with suspected pulmonary embolism. J Thromb Haemost. 2004;2(8):1247‐1255. 15304025 10.1111/j.1538-7836.2004.00790.x

[17] Wicki J , Perneger T , Junod A , Bounameaux H , Perrier A . Assessing clinical probability of pulmonary embolism in the emergency ward: a simple score. Arch Intern Med. 2001;161(1):92‐97. 11146703 10.1001/archinte.161.1.92

[18] Nazareth D , Walshaw M . A review of renal disease in CF. J Cyst Fibros. 2013;12(4):309‐317. 23618617 10.1016/j.jcf.2013.03.005

[19] Wells P , Anderson D , Rodger M , et al. Evaluation of D‐dimer in the diagnosis of suspected deep‐vein thrombosis. N Engl J Med. 2003;345(13):1227‐1235. 10.1056/NEJMoa023153 14507948

[20] Wells P , Anderson D , Bormanis J , et al. Value of assessment of pretest probability of deep‐vein thrombosis in clinical management. Lancet. 1997;350(9094):1795‐1798. 9428249 10.1016/S0140-6736(97)08140-3

[21] Raja A , Greenberg J , Qaseem A , et al. Evaluation of patients with suspected acute pulmonary embolism: best practice advice from the clinical guidelines committee of the American College of Physicians. Ann Intern Med. 2015;163(9):701‐711. 26414967 10.7326/M14-1772

[22] Posadas‐Martínez M , Vázquez FJ , Giunta D , Waisman G , de Quirós F , Gándara E . Performance of the Wells score in patients with suspected pulmonary embolism during hospitalization: a delayed‐type cross sectional study in a community hospital. Thromb Res. 2014;133(2):177‐181. 24342535 10.1016/j.thromres.2013.11.018

[23] Silveira P , Ip I , Goldhaber S , Piazza G , Benson C , Khorasani R . Performance of Wells score for deep vein thrombosis in the inpatient setting. JAMA Intern Med. 2015;175(7):1112‐1117. 25985219 10.1001/jamainternmed.2015.1687

[24] van der Hulle T , Cheung W , Kooji S , et al. Simplified diagnostic management of suspected pulmonary embolism (the YEARS study): a prospective, multicentre, cohort study. Lancet. 2017;390(10091):289‐297. 28549662 10.1016/S0140-6736(17)30885-1

[25] Kearon C , de Wit K , Parpia S , et al. Diagnosis of PE with D‐dimer adjusted to clinical probability. NEJM. 2019;381(22):2125‐2134. 31774957 10.1056/NEJMoa1909159

[26] Van Es N , van der Hulle T , van Es J , et al. Wells rule and D‐dimer testing to rule out pulmonary embolism: a systematic review and individual‐patient data meta‐analysis. Ann Intern Med. 2016;165(4):253‐261. 27182696 10.7326/M16-0031

